# TRUST Technique for Neurointervention: A Promising Alternative for Complex Cases

**DOI:** 10.2174/0115672026291503240105093155

**Published:** 2024-01-17

**Authors:** Xinzhao Jiang, Peng Wang, Fang Liu, Huadong Wu, Peng Jiang, Ruozhen Yuan, Sheng Zhang, Zongjie Shi

**Affiliations:** 1 Center for Rehabilitation Medicine, Department of Neurology, Zhejiang Provincial People’s Hospital (Affiliated People's Hospital, Hangzhou Medical College), Hangzhou, 310014, Zhejiang, China

**Keywords:** Endovascular treatment, ischemic stroke, neurointervention, neurology, neurovascular diseases, complications

## Abstract

**Background:**

Neurointervention *via* Transradial Access (TRA) is becoming increasingly popular as experience with this technique increases. However, approximately 8.6–10.3% of complex TRA cases are converted to femoral access due to a lack of support or radial artery spasm. This study aimed to assess the efficacy and safety of the TRUST (trans-radial coaxial catheter technique using a short sheath, Simmons catheter, and Tethys intermediate catheter) technique in interventional procedures *via* TRA.

**Methods:**

This was a single-center retrospective analysis of 16 patients admitted to our institute between January 2023 to May 2023 to undergo endovascular interventions with the TRUST technique *via* the TRA.

**Results:**

The mean age of the study population was 63.8 years, and 62.5% were male (10/16). The most common procedure was intracranial atherosclerotic stenosis (93.75%, 15/16). All procedures were performed successfully, and the most common procedures in our cohort were ballooning (50.0%, 8/16), stenting (18.75%, 3/16), and both procedures combined (31.25%, 1/16). All procedures were performed using the TRA, and the distal and proximal radial arteries were used for access in 31.35% (5/16) and 68.75% (11/16) of the cases, respectively. Technical success was achieved in all patients and most cases demonstrated mTICI ≥2b recanalization (93.75%, 15/16). In this case, no major access-site complications occurred.

**Conclusion:**

The TRUST technique is technically safe and feasible and had a high technical success rate and low complication rate in our study. These results demonstrate that the TRUST technique is a promising alternative for patients undergoing complex neurointerventions.

## 1 INTRODUCTION

Favorable evidence demonstrating a reduction in access site complications, in addition to improved patient preference and cost compared with traditional transfemoral access following the Trans-Radial Approach (TRA), has increased the adoption of TRA in the coronary and peripheral procedure [1-9]. Therefore, neurointervention *via* the TRA has become more popular in recent years. Many studies have shown that TRA for neuro-interventional procedures is associated with a lower risk of access site complications than the transfemoral access approach (TFA) [9-12]. Additionally, postprocedural bed rest is not required, facilitating early ambulation and discharge following procedures that do not require hospital admission. These benefits are increasingly promoting the use of TRA in neurointervention, resulting in the rapid and safe development of numerous techniques [13-15]. However, a proportion of neurointerventional procedures are performed using the TFA rather than the TRA because of radial artery access failure, anatomical variability, and lack of a catheter system [16, 17]. The lack of catheter systems specifically designed for TRA is a primary challenge [18]. Currently, several methods are being developed. Although TRA can be performed using the TFR catheter systems, the smaller size of the radial artery and different trajectories into the great vessels from the arm render the femoral catheter design suboptimal [16, 17].

Therefore, high-quality TRA equipment can overcome the main barriers to TRA adoption and broaden TRA development [18]. Two studies showed that the first catheter [17] and the Zoom RDL Radial Access System [16] are technically feasible and effective in achieving complex neurointerventional procedures with low complication rates. However, these studies were conducted on patients from the United States. Therefore, a suitable TRA device is required for Chinese patients.

In this study, we present our preliminary experience with the TRUST (trans-radial coaxial catheter technique using a short sheath, Simmons catheter, and Tethys intermediate catheter) system for neuro-interventional procedures using TRA in Chinese patients, focusing on its technical feasibility and safety. This study thereby aimed to develop a promising alternative for complex neurointervention cases.

## 2 MATERIALS AND METHODS

This study retrospectively reviewed consecutive patients who underwent neuro-interventional procedures using the TRUST technique at our center between January 2023 and May 2023. This retrospective study was approved by the Institutional Review Board of our institute. Given the retrospective nature of this study, the requirement for written informed consent was waived.

In total, we included 16 patients who were treated by a single operator using the TRUST technique. The inclusion criteria were as follows: (1) Age ≥18 years; (2) Intracranial atherosclerotic stenosis;(3) underwent procedures involving the TRUST technique. The exclusion criteria were as follows: (1) failed radial access and (2) conversion to a guide catheter or TFA during the operation. Finally, the following data were recorded: procedural details, radiographic findings, and hospitalization events. The primary outcome was access success, defined as access to the target vessel and finishing operation [16]. The secondary outcomes were procedural complications and in-hospital mortality. Procedural complications included access site- and device-related complications.

### 2.1 TRUST System

The TRUST system comprises three catheters, a 6F Tethys intermediate catheter (ACHEVA, Shanghai, China), a 5F Simmons two catheter (Terumo, Tokyo, Japan) and a 6F short sheath (Terumo, Tokyo, Japan). The 6F Tethys intermediate catheter measured 105, 115, and 125 cm and was designed for peripheral or intracranial vascular interventions through radial or femoral artery access and has the following advantages: (1) flexible and supportive segments, (2) larger inner diameter, and (3) smaller outer diameter. These advantages suggest that intracranial vascular interventions can be performed *via* the radial arteries.

### 2.2 Process for the Use of the TRUST System

**Fig. (1):**
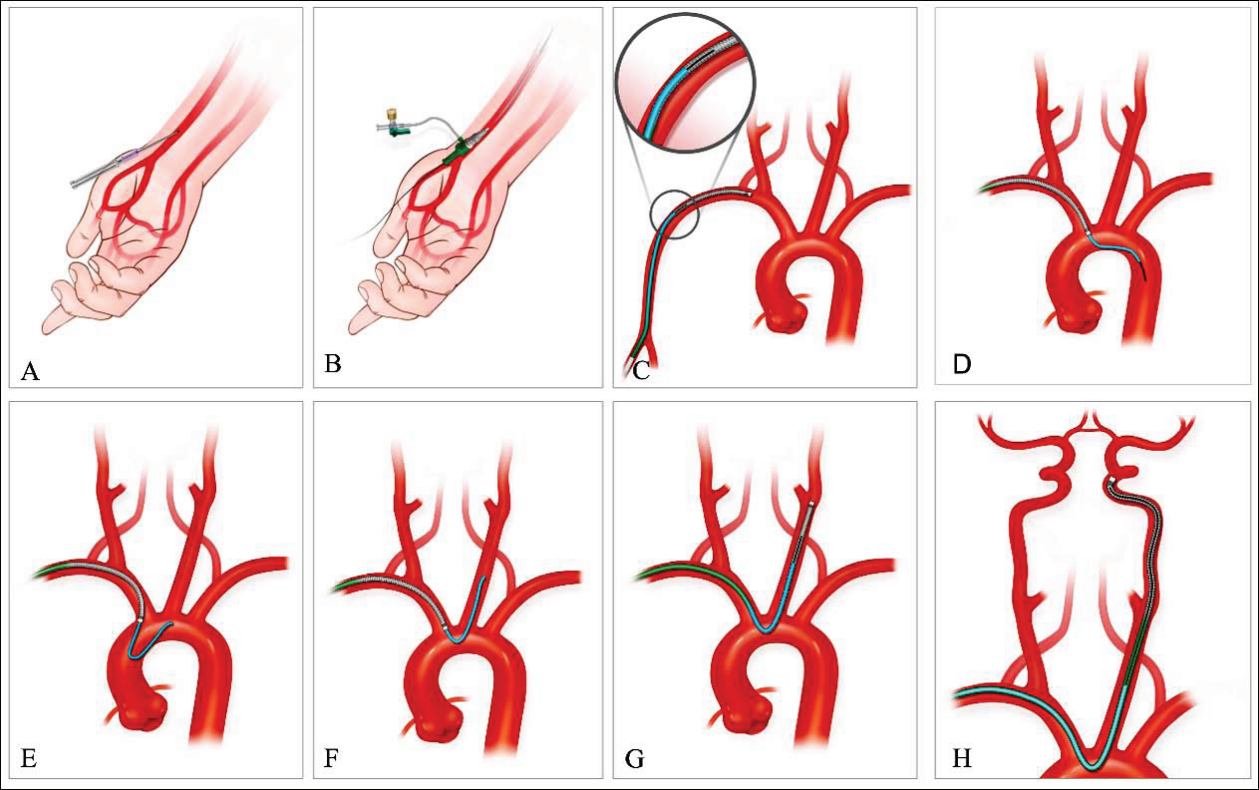
Illustration demonstrating the process of TRUST technique: (**A**), cannulating radial artery; (**B),** 6F radial sheaths is introduced into the radial artery; (**C**), Simmons two catheter was used in combination with a Tethys intermediate catheter were positioned in the subclavian artery; (**D**), Simmons two catheter was used in combination with a Tethys intermediate catheter were delivered to the descending aorta; (**E**), loop of the Simmons two catheter was formed in the ascending aorta; (**F**), Simmons two catheter was catheterize into target vessel; (**G**), Tethys intermediate catheter is introduced into target vessel; (**H**), Tethys intermediate catheter into the distal cervical segment of the internal carotid artery.

The radial artery was selected as the primary access point in cases where we used the TRUST system, except when the operation was difficult (radial loop, high radial takeoff, and type III aortic arch) using the right radial artery, in which case, we usually decided to use the left radial artery. It is also easier for neurointerventionalists to operate on the right side of the patient. Barbeau's and Allen's tests are not performed as preoperative collateral tests because they cannot accurately assess the vascular collateral patency of the hand [19]. Ultrasound or radial angiography was performed before surgery in all cases to confirm that the used radial artery had a diameter ≥2.0 mm(6F). A 6F radial sheath (Terumo, Tokyo, Japan) was introduced into the radial artery using the Seldinger technique [20]. Verapamil 2.5 mg, nitroglycerin 200μ, and heparin 3000 units were infused into the artery *via* the radial sheaths to reduce the risk of radial artery spasm/occlusion (RAS/RAO) [21] (Figs. **1A** and **1B**). Subsequently, to identify the radial artery track or anatomical variants, radial artery angiograms and roadmaps were performed before introducing guidewires [22, 23]. Based on this roadmap, a guidewire was introduced *via* the 6F radial sheath. Two catheters were used in combination with a Tethys intermediate catheter and delivered to the descending aorta under the guidance of a guidewire (Figs. **1C** and **1D**). The Tethys intermediate catheter was catheterized into the target vessel using the descending aorta looping formation (Fig. **1E** and **1F**) [24]. Next, a Tethys intermediate catheter was used to carefully insert the proximal target vessel and obtain a roadmap for visualizing the intracranial vasculature (Fig. **1G**). A Tethys intermediate catheter was advanced into the distal cervical segment of the internal carotid artery (ICA) (Fig. **1H**). Finally, the Simmons two catheter and guidewires were removed.

## 3 RESULTS

### 3.1 Baseline Characteristics of Patients

Table **1** presents the baseline characteristics of the patients who underwent endovascular therapy. In this study, intracranial atherosclerotic stenosis was the most common type (93.75%, 15/16). The most common procedures in our cohort were ballooning (50.0%, 8/16), stenting (18.75%, 3/16), and both procedures simultaneously (31.25%, 1/16). All procedures were performed using the TRA, and the distal and proximal radial arteries were used for access in 31.35% (5/16) and 68.75% (11/16) of the cases, respectively. Of all the patients, 6.25% (1/16) had access to the left radial artery. The radial artery diameter was >2 mm in 93.72% (15/16) cases. The TRUST system was used in all the cases.

### 3.2 Procedural Outcomes and Complications

Table **2** shows the procedural outcomes and complications. All patients achieved procedural and technical success using the TRUST system. Further, the majority of the cases (93.75%, 15/16) demonstrated mTICI ≥2b recanalization. In this case, no major access-site complications occurred. However, other procedure-related complications were as follows: [1] One patient with severely stenotic MCA experienced headache after balloon placement. Hyperperfusion Syndrome (HPS) was one of the most devastating complications associated with intracranial angioplasty and stenting, and Endovascular Thrombectomy (EVT) [25]. To manage HPS, strict control of blood pressure effectively relieves the patient’s headaches [2]. One patient showed visual disturbances after stenting, which may have been associated with contrast [26]. Dexamethasone and sodium chloride were injected into the patients to facilitate the elimination of the contrast agent. The patient showed slow improvement in visual function, with complete recovery within 5 days [3]. One patient with nonacute intracranial ICA occlusion developed chemosis after recanalization, which was related to intracranial hemodynamic changes that may increase eye blood flow as the ICA opening exceeds the metabolic demands of the eyes [4]. One patient experienced numbness in the right hand after balloon placement, and the MRI demonstrated that the left cerebral tissue had a small infarct, which was considered to be an embolus to the distal territory. All patients were safely discharged from the hospital after the procedure.

## 4 DISCUSSION

The results of this study showed the safety and feasibility of the TRUST system for neurointerventional procedures *via* TRA, emphasizing a high technical Access success rate and a low complication rate of the TRUST system.

### 4.1 Key of TRUST System

The key component of the TRUST system is the Tethys Intermediate Catheter, which is characterized by the following features: (1) The proximal portion of the Tethys intermediate catheter is supported by a full coil combined with braiding technology, which improves proximal support and achieves better push ability and torque transmission for distal navigation. The proximal portion of the Tethys intermediate catheter has a soft 16-centimeter section which improves proximal compliance and tortuous vessels (2). A larger inner diameter is beneficial for large-bore aspiration and enables the passage of multiple devices (3). The proximal portion of the Tethys intermediate catheter has a 25° pre-shape, which directs the guidewire and facilitates target vascular selection. (4). Tethys intermediate catheter has a hydrophilic coating on its surface that can potentially reduce the risks related to catheter manipulation and entrapment in the radial artery. Previous studies have demonstrated that the lack of a hydrophilic coating may be related to an improved risk of RAS, Radial Artery Occlusion (RAO) and patient’s discomfort [27]. Severe RAS can contribute to hand ischemia, clot formation, and infections. In our study, no RAS, symptomatic RAO, or catheter entrapment was observed, which may be attributed to the hydrophilic coating.

### 4.2 Catheter Function

The neurointerventional procedure *via* the TRA still presents significant challenges compared with the TFA, owing to anatomical differences. Examples include the radial loop, large-diameter aortic arch, and acute subclavian vertebral angle. This reduces the access success rate and increases the risk of complications when using TRA. Understanding these anatomical differences is crucial for the device selection.

To reach the distal ICA or MCA branch, the proximal portion of the Tethys intermediate catheter has a soft 16-centimeter section, which can improve proximal compliance through tortuous vessels. Meanwhile, a full coil combined with braided technology can reduce the likelihood of catheter prolapse into the arch. Multiple stents and balloons are compatible with the Tethys intermediate catheter. This versatility enables the use of the Tethys intermediate catheter in a variety of procedures and target vessels. However, the profiles of catheter support need to be adjusted according to the disease pathology, anatomical variants, and experience of the neurointerventionalist.

### 4.3 Outcomes and complications

The rate of crossover from the TRA to TFA is approximately 8% when femoral-designed catheters are used [10, 12, 28-30]. Recently, Morsi *et al*. adapted the Zoom RDL radial access system for neurointervention *via* TRA [16], finding that the crossover rate was approximately 6.9%, which is lower than that of the traditional femoral-designed catheters. Abecassis *et al*. improved the radial access system for neurointerventions *via* the TRA [17]; in this study, the crossover rate from the TRA to TFA was decreased to 3.9% using Rist's radial access system. In our cohort, all patients were successfully treated with TRA. Successful recanalization of a patient with nonacute intracranial vessel occlusion demonstrated the stable support capability of the TRUST system.

RAO is a common complication of the TRA. Previous interventional cardiology studies have reported asymptomatic RAO rates between 1% and 10% [14, 15]. Access site complications were not observed in our cohort, which may be due to hydrophilic coating on Tethys intercatheter surface and consistent use of ‘cocktails’ to reduce the risk of RAS/ RAO. However, other complications unrelated to the TRUST system included hyperperfusion syndrome (1/16), visual disturbance (1/16), chemosis (1/16), and distal thrombus migration (1/16). Finally, all patients with complications recovered completely before discharge.

### 4.4 Future Directions and Limitations

Based on our previous experience with the TRUST system, the Tethys intermediate catheter provides a repertoire of advantages ranging from distal navigation capabilities and maneuverability between more than one target vessel, as demonstrated in our case illustration, to a strong proximal support system, which may play an important role in a variety of procedure types. However, the retrospective nature of the study and the relatively small number of patients are some of the most important factors limiting the clinical significance of the current results. In the future, researchers will be able to build on the TRUST system and further optimize it to treat more complex neuro interventions.

## CONCLUSION

The TRUST technique was technically feasible and safe, had a high technical success rate, and a low complication rate in our study. These results demonstrate that the TRUST technique is a promising alternative for patients undergoing complex neurointerventions.

## Figures and Tables

**Table 1:** Baseline characteristics of patients.

Baseline Characteristics of Patients (n=16)	
**Age, Years (Mean)**	63.8
**Sex**	
Male	10(62.5%)
Female	6(37.5%)
**Pathology**	
Intracranial atherosclerotic stenosis	15(93.75%)
Non-acute intracranial vessel occlusion	1(6.25%)
**Type of Procedure**	
Balloon	8(50.0%)
Stenting	3(18.75%)
Ballon and stenting	5(31.25%)
**Access Site**	
Right proximal radial	10(62.5%)
Right distal radial	5(31.35%)
Left proximal radial	1(6.25%)
Left distal radial	0(0.00%)
**Radial Diameter>2mm**	
Yes	15(93.75%)
No	1(6.25%)
**Target Vessel for Intervention**	
Right intracranial ICA	3(18.75%)
Left intracranial ICA	4(25.0%)
Right MCA	7(43.75%)
Left MCA	0(0.00%)
Right ACA	0(0.00%)
Left ACA	1(6.25%)
Right vertebral artery	1(6.25%)
Left vertebral artery	0(0.00%)

**Table 2:** Rates of success and complication.

Rates of Success and Complication (n=16)	
**Technical Access Success**	
Yes	16(100.0%)
No	0(0.00%)
**Modified TICI Score on Final Angiogram**	
≥2b	15(93.75%)
< 2b	1(6.25%)
**Access Site Complications**	
Yes	16(100.0%)
No	0(0.00%)
**Other Procedure-related Complications**	
No	12(75.0%)
Hyperperfusion syndrome (HPS)	1(6.25%)
Contrast-induced encephalopathy (CIE)	1(6.25%)
Chemosis	1(6.25%)
Emboli to distal territory	1(6.25%)
**RAS**	0(0.0%)
**Length of stay, days (mean)**	12

## Data Availability

The datasets used and/or analyzed during the current study are available from the corresponding author on a reasonable request.

## LIST OF ABBREVIATIONS

RAO: Radial Artery Occlusion
TFA: Transfemoral Access Approach
TRA: Transradial Access

## STANDARDS OF REPORTING

STROBE guidelines were followed.
